# Hydrophobicity drives the systemic distribution of lipid-conjugated siRNAs via lipid transport pathways

**DOI:** 10.1093/nar/gky1232

**Published:** 2018-12-08

**Authors:** Maire F Osborn, Andrew H Coles, Annabelle Biscans, Reka A Haraszti, Loic Roux, Sarah Davis, Socheata Ly, Dimas Echeverria, Matthew R Hassler, Bruno M D C Godinho, Mehran Nikan, Anastasia Khvorova

**Affiliations:** 1 RNA Therapeutics Institute, University of Massachusetts Medical School, Worcester, MA, USA; 2 Department of Molecular Medicine, University of Massachusetts Medical School, Worcester, MA, USA

## Abstract

Efficient delivery of therapeutic RNA beyond the liver is the fundamental obstacle preventing its clinical utility. Lipid conjugation increases plasma half-life and enhances tissue accumulation and cellular uptake of small interfering RNAs (siRNAs). However, the mechanism relating lipid hydrophobicity, structure, and siRNA pharmacokinetics is unclear. Here, using a diverse panel of biologically occurring lipids, we show that lipid conjugation directly modulates siRNA hydrophobicity. When administered *in vivo*, highly hydrophobic lipid-siRNAs preferentially and spontaneously associate with circulating low-density lipoprotein (LDL), while less lipophilic lipid-siRNAs bind to high-density lipoprotein (HDL). Lipid-siRNAs are targeted to lipoprotein receptor-enriched tissues, eliciting significant mRNA silencing in liver (65%), adrenal gland (37%), ovary (35%), and kidney (78%). Interestingly, siRNA internalization may not be completely driven by lipoprotein endocytosis, but the extent of siRNA phosphorothioate modifications may also be a factor. Although biomimetic lipoprotein nanoparticles have been explored for the enhancement of siRNA delivery, our findings suggest that hydrophobic modifications can be leveraged to incorporate therapeutic siRNA into endogenous lipid transport pathways without the requirement for synthetic formulation.

## INTRODUCTION

For over a decade, the underlying obstacle preventing the widespread clinical use of small interfering RNA (siRNA)-based therapies has been efficient and safe *in vivo* delivery. siRNAs are large (∼14 kDa), polyanionic macromolecules that require extensive modifications to improve their inherently-poor pharmacological properties (e.g. plasma half-life of <5 min before renal excretion) (1,2). Lipid- and polymer-based nanoparticles, which are effective transfection agents *in vitro*, can prolong circulation time and improve stability and bioavailability *in vivo*. However, nanoparticle delivery is typically limited to clearance organs with fenestrated and/or discontinuous endothelium (e.g. liver, spleen and certain tumors). Moreover, nanoparticles can interact with opsonin proteins that enhance clearance by macrophage phagocytosis.

Molecular-scale delivery of siRNAs with small targeting ligands, cell-penetrating peptides, or lipid conjugates may be a simple and effective alternative to nanocarrier-based methods. The most clinically-advanced siRNA conjugate, trivalent *N*-acetylgalactosamine (GalNAc)-siRNA, binds to the asialoglycoprotein receptor on hepatocytes with high selectivity, and triggers potent and durable (∼6 months) gene silencing in patients (3,4). The second major class of molecular siRNA conjugates are lipids, which have been shown to enhance circulation time and promote local and systemic delivery and efficacy. Cholesterol-modified siRNA, one of the first reported lipid conjugates, silences liver apolipoprotein B (ApoB) expression at high doses (50 mg kg^−1^), while α-tocopherol and fatty acid conjugates have also shown potential for liver delivery and gene silencing (5–7). Productive internalization of cholesterol-conjugated siRNAs hinges on its interactions with circulating lipoproteins and tissue receptors (6). However, the relationship between the chemical composition of lipid conjugates and siRNA pharmacology has not been well characterized.

In order to define design parameters for optimizing therapeutic oligonucleotide delivery, we investigated the influence of structurally diverse lipids on conjugate-mediated siRNA biodistribution, efficacy, and safety *in vivo*. These studies were enabled by the use of a clinically-validated, chemically-modified siRNA scaffold that is nuclease resistant and metabolically stable (8). Here, we demonstrate that lipid conjugation modulates the hydrophobicity of hsiRNA (hydrophobically modified siRNAs). We present evidence suggesting that oligonucleotide hydrophobicity governs pharmacokinetic behavior by driving selective, *in situ* incorporation into endogenous lipoprotein pathways. Lipid modification also enables potent siRNA-mediated mRNA silencing in lipoprotein receptor-enriched tissues, including liver, adrenal gland, ovary and kidney. These data suggest that hydrophobicity likely directs the rank-order tissue distribution of lipidic oligonucleotide conjugates that may otherwise be intended to be specifically receptor- or cell-targeting.

## MATERIALS AND METHODS

### Oligonucleotide synthesis

Oligonucleotides were synthesized using standard and modified (2′-fluoro, 2′-*O*-methyl) phosphoramidite, solid-phase synthesis conditions using a MerMade 12 (BioAutomation) and Expedite ABI DNA/RNA synthesizer (ABI 8909). Oligonucleotides were removed from controlled pore glass (CPG), deprotected, and HPLC purified as described previously (8–10). Ion exchange was performed on purified oligonucleotides using a Hi-Trap cation exchange column. The identity of oligonucleotides was established by LC-MS analysis (Waters Q-TOF premier). Relative degree of hydrophobicity of sense strands was assayed by reverse-phase HPLC (Waters Symmetric 3.5 μm, 4.6 × 75 mm column) using a 0–100% gradient over 15 min at 60°C with 0.1% TEAA in water (eluent A) and 100% acetonitrile (eluent B). Peaks were monitored at 260 nm.

### Oligonucleotide delivery

HeLa cells were plated in DMEM containing 6% FBS at 10,000 cells per well in 96-well tissue culture plates. hsiRNA was diluted to twice the final concentration in OptiMEM (Gibco), and mixed 1:1 with Lipofectamine RNAiMAX Transfection Reagent (Invitrogen) (final transfection reagent concentration = 0.3 μl/25 μl/well). 50 μl-diluted hsiRNA was added to 50 μl of cells, resulting in a final concentration of 3% FBS. Cells were incubated for 72 hours at 37°C and 5% CO_2_. For all *in vivo* studies, lipid-hsiRNAs were delivery without a transfection reagent.

### mRNA quantification

mRNA was quantified using the QuantiGene 2.0 Assay (Affymetrix) as described previously (8). Briefly, cells were lysed in 250 μl-diluted lysis mixture with Proteinase K (Affymetrix) for 30 min at 55°C prior to mRNA quantification. Tissue punches (∼5 mg) were homogenized in 300 μl of Homogenizing Buffer (Affymetrix) with proteinase K in 96-well plate format using a TissueLyser II (Qiagen). This method is described in detail in Coles *et al* (11).

### Mouse studies

All animal procedures were approved by the University of Massachusetts Medical School Institutional Animal Care and Use Committee (IACUC, protocol number A-2411). Mice (FVB/NJ) were 6–10 weeks of age at the time of experiments. All animals were kept on a 12-h light/dark cycle in a pathogen-free facility with food and water *ad libitum*. For imaging and mRNA-silencing studies, mice were injected subcutaneously (SC, interscapular, between shoulders) or intravenously (IV, via tail vein) with 10–20 mg kg^−1^ of Cy3-labeled oligonucleotides (see figure captions for details). After 4 h-1 week, mice were deeply anesthetized with 0.1% Avertin and transcardially perfused with a 4% paraformaldehyde solution in phosphate-buffered saline, pH 7.2. Tissues were collected and incubated overnight at 4°C in either 10% formalin for subsequent imaging or RNALater (Ambion) for subsequent mRNA quantification. Tissues were processed as described previously (8).

### Fluorescence microscopy experiments

All fluorescence images were acquired with a Leica DM5500 microscope fitted with a DFC365 FX fluorescence camera. After image acquisition, images were exported in 16-bit TIFF format from the Leica LAS X software and processed in Fiji v1.51n (12) to quantify the kinetics of oligo uptake into cells. Briefly, the freehand tool was used to manually select individual cells and the mean fluorescence intensity was obtained by using the ‘Measure’ function. Background fluorescence was corrected for by measuring the mean fluorescence of an area with no cells and subtracting this value from the cellular fluorescence intensity. The histology of the liver is well defined, and cell structure features can be used to identify different cell types, which are confirmed by immunostaining (13).The major cell types in liver—hepatocytes (∼52%), Kupffer cells (∼18%), and endothelial cells (∼22%)—are easily defined by their distinct morphological features. Hepatocytes are large cells and possess large nuclei, most of which are tetraploid. Endothelial cells are smaller, elongated cells that surround vessels. Kupffer cells are the smallest cell type and are characterized by dense nuclei.

### Peptide nucleic acid (PNA) hybridization assay

Tissue accumulation of lipid-conjugated hsiRNAs was quantified as described previously (8,14). Briefly, tissues were lysed in MasterPure tissue lysis solution (EpiCentre) in the presence of proteinase K (2 mg/ml) (Invitrogen) using a TissueLyser II (Qiagen) (∼10 mg tissue per 100 μl lysis solution). Sodium dodecyl sulfate (SDS) was precipitated with KCl (3 M) and pelleted at 5000 × g. hsiRNAs presented in the cleared supernatant was hybridized to a Cy3-labeled PNA that was fully complementary to guide strand (PNABio) and injected on a DNAPac PA100 anion exchange column (Thermo Fisher). Cy3 fluorescence was monitored at 570 nm.

### Lipoprotein size exclusion chromatography

For lipoprotein profiling, mice were injected intravenously with 20 mg kg^−1^ of Cy3-labeled oligonucleotides. After 15 minutes, whole mouse blood (∼500 μl) was collected in a sterile EDTA-coated tube following cheek incision with a lancet. Samples were spun at 10,000 RPM for 10 minutes at 4°C. 50 μl of serum was directly injected on Superose 6 Increase 10/300 size exclusion column (GE Healthcare). Oligonucleotide migration was monitored by Cy3 fluorescence at 570 nm, and lipoprotein protein content was monitored by absorbance at 280 nm.

### Statistical analysis

Data were analyzed using GraphPad Prism 6 software. IC_50_ curves were fitted using log(inhibitor) versus response—variable slope (four parameters). For Figure 4, statistics were calculated using one-way ANOVA with Tukey's test for multiple comparisons, with significance calculated relative to saline control-injected animals. For Figure 5, statistics were calculated using an unpaired, two-tailed t-test.

## RESULTS

### Design and synthesis of structurally-diverse lipid-hsiRNA conjugates

**Figure 1.**
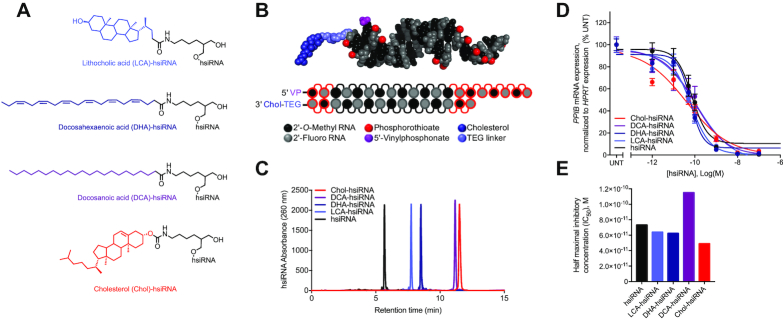
Synthesis and biophysical characterization of lipid-hsiRNA conjugates. (**A**) Chemical structures of lipid-hsiRNA conjugates. (**B**) Modification pattern and molecular model of lipid-hsiRNAs. (**C**) HPLC traces of lipid-hsiRNAs following reverse phase column chromatography. (**D**) HeLa cells were incubated with *PPIB*-targeting hsiRNAs at concentrations shown for 72 h. *PPIB* mRNA levels were measured using QuantiGene (Affymetrix), normalized to housekeeping *HPRT1* (hypoxanthine phosphoribosyltransferase 1) mRNA levels, and presented as percent of untreated control (*n* = 3, mean ± SD). UNT – untreated cells. (**E**) Plotted IC_50_ values determined from the best-fit curves in (D).

To evaluate the impact of lipid conjugation on the pharmacological properties of oligonucleotides, we synthesized a panel of structurally diverse conjugates using a hydrophobically-modified siRNA scaffold, termed hsiRNA (Figure 1A, 1B) (9,10,15). This scaffold consists of a 20-nucleotide (nt) antisense (guide) strand and a 15-nt sense (passenger) strand, and contains alternating 2′-*O*-methyl and 2′-fluoro sugar modifications (16). The 5′-end of the antisense strand bears a terminal (*E*)-vinylphosphonate modification that increases siRNA tissue accumulation, extends the duration of silencing activity, and shields oligonucleotides from 5′-to-3′ exonucleases (14,17–19). The terminal nucleotide backbones are fully phosphorothioated to further inhibit exonuclease-mediated degradation and to promote cellular internalization (20). These extensive modifications are essential for evaluating conjugate-mediated delivery *in vivo* because partially-modified or unmodified siRNAs are rapidly degraded and cleared (8).

Previous studies have identified the 3′-end of the sense strand as an optimal position for conjugate attachment, with minimal effect on siRNA-RISC (intracellular RNA-induced silencing complex) loading (10,21). Therefore, we designed cholesterol (Chol), lithocholic acid (LCA), docosahexaenoic acid (DHA), and docosanoic acid (DCA) conjugates to attach through a commercially available carbon-based linker to the 3′-end of the sense strand via an amide bond (Figure 1A). hsiRNA conjugates were synthesized on a functionalized solid support bearing each individual lipid moiety using standard solid-phase oligonucleotide synthesis and deprotection protocols (see Methods). Synthesized oligonucleotides were then purified and characterized by high-performance liquid chromatography (HPLC) and liquid chromatography–mass spectrometry (LC–MS), respectively. All oligonucleotide sequences and chemical modification patterns used in this study are reported in Supplementary Table S1.

To evaluate the impact of lipid conjugation on hsiRNA hydrophobicity, we analyzed Chol-hsiRNA, DCA-hsiRNA, DHA-hsiRNA, LCA-hsiRNA and unconjugated hsiRNA by reverse-phase HPLC and measured retention times (Figure 1C). Here, a longer retention time correlates with greater affinity for the hydrophobic (C18) stationary phase. Unconjugated hsiRNAs eluted relatively quickly (5.7 min), followed by LCA-hsiRNA (7.7 min), DHA-hsiRNA (8.5 min), DCA-hsiRNA (11.1 min), and Chol-hsiRNA (11.5 min). This result indicates that DCA- and Chol-hsiRNAs are more hydrophobic than DHA-, LCA-, or unconjugated hsiRNAs. The nature of the lipid conjugate has a clear impact on overall hydrophobicity. In general, an increase in length causes an increase in overall hydrophobicity. By contrast, an increase in the degree of unsaturation makes compounds less hydrophobic. Retention times did not directly correlate with the predicted partition coefficients of the free lipids (Supplementary Table S2), suggesting that lipid orientation and conjugation position influence overall siRNA hydrophobicity.

To assess the effect of lipid conjugation on RISC loading and siRNA silencing efficiency *in vitro*, we transfected each lipid-hsiRNA into HeLa cells and measured mRNA levels of a well-validated housekeeping gene, cyclophilin B (*PPIB*) (Figure 1D). Calculated half-maximal inhibitory concentrations (IC_50_s) ranged from 4.9 × 10^−11^ to 1.2 × 10e^−10^ M, confirming that conjugation at the 3′-end of the sense strand does not substantially affect RNAi potency (Figure 1E). In summary, we successfully synthesized a panel of lipid-hsiRNA conjugates with varying degrees of hydrophobicity that retain gene silencing activity *in vitro*.

### Lipid conjugation reduces hsiRNA kidney exposure and promotes broad biodistribution

**Figure 2.**
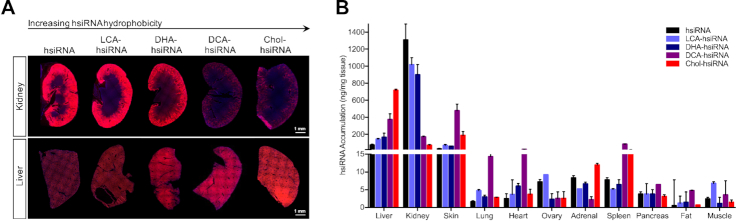
Systemic biodistribution and tissue accumulation of lipid-hsiRNA conjugates. Biodistribution of lipid-hsiRNAs 48 h after a single, subcutaneous injection (*n* = 3 mice, 20 mg kg^−1^). (**A**) Kidney and liver distribution of lipid-conjugated hsiRNAs. Cy3-labeled lipid-hsiRNAs (red), nuclei stained with DAPI (blue). (**B**) Guide strand quantification of Cy3-labeled lipid-hsiRNAs by a PNA hybridization-based assay. Data presented as mean ± SD.

To evaluate the biodistribution of each lipid-hsiRNA conjugate *in vivo*, we administered Cy3-labeled lipid-hsiRNAs into mice by a single, subcutaneous injection (*n* = 2, 20 mg kg^−1^) and measured fluorescence distribution after 48 h. As lipid-hsiRNA hydrophobicity increased, we observed a clear, progressive reduction in kidney accumulation and an increase in liver retention (Figure 2A). We confirmed this result using a peptide-nucleic acid (PNA) hybridization assay, which measures hsiRNA antisense strand concentration in tissue lysate by HPLC. Unconjugated hsiRNAs were subject to acute renal clearance after administration and reached peak tissue concentrations of ∼1300 ng hsiRNA/mg kidney (Figure 2B). As lipid-hsiRNA hydrophobicity increased, kidney accumulation decreased over 18-fold (peak concentration of ∼70 ng hsiRNA/mg kidney for Chol-hsiRNA). Our findings agree with previous pharmacokinetic analyses of lipid-conjugated hsiRNAs, which found that the initial distribution half-life of Chol-hsiRNA was 33–35 min, but was only 15–17 min for unconjugated hsiRNA (22). Moreover, the total exposure over time (area under the curve; AUC) increased >10-fold for Chol-hsiRNA compared to unconjugated hsiRNA (22). Unlike the kidney, oligonucleotide concentrations in the liver increased with increasing hydrophobicity of the lipid conjugate (∼75 ng mg^−1^ for unconjugated hsiRNA up to ∼715 ng mg^−1^ for Chol-hsiRNA). Lipid conjugation also enabled delivery beyond the liver and kidney, supporting conjugate-mediated siRNA accumulation in lung, heart, adrenal gland, spleen, adipose tissue, and femoral muscle (Figure 2B).

### Hydrophobicity governs the interaction between lipid-hsiRNAs and serum proteins

To investigate potential mechanism(s) governing the association between lipid-siRNA hydrophobicity and biodistribution, we measured the affinity of lipid-siRNAs toward plasma constituents both *in vitro* and *in vivo*. Binding to albumin, a permissive host for both endogenous and exogenous ligands (23), has been proposed to improve the pharmacokinetics of lipid-conjugated oligonucleotides (7). Thus, we measured the affinity of Cy3-labeled lipid-hsiRNAs and bovine serum albumin (BSA) *in vitro* using native gel electrophoresis (Supplementary Figure S1). Unmodified hsiRNA did not associate with BSA to any measureable degree. However, all lipid-hsiRNA conjugates formed stable complexes with BSA, and exhibited dose-dependent migration shifts with increasing albumin concentration. We observed a direct correlation between hsiRNA hydrophobicity and BSA binding, with DCA-hsiRNA and Chol-hsiRNA exhibiting the highest affinities (*K*_d_ ∼ 120 and 164 μM, respectively).

**Figure 3.**
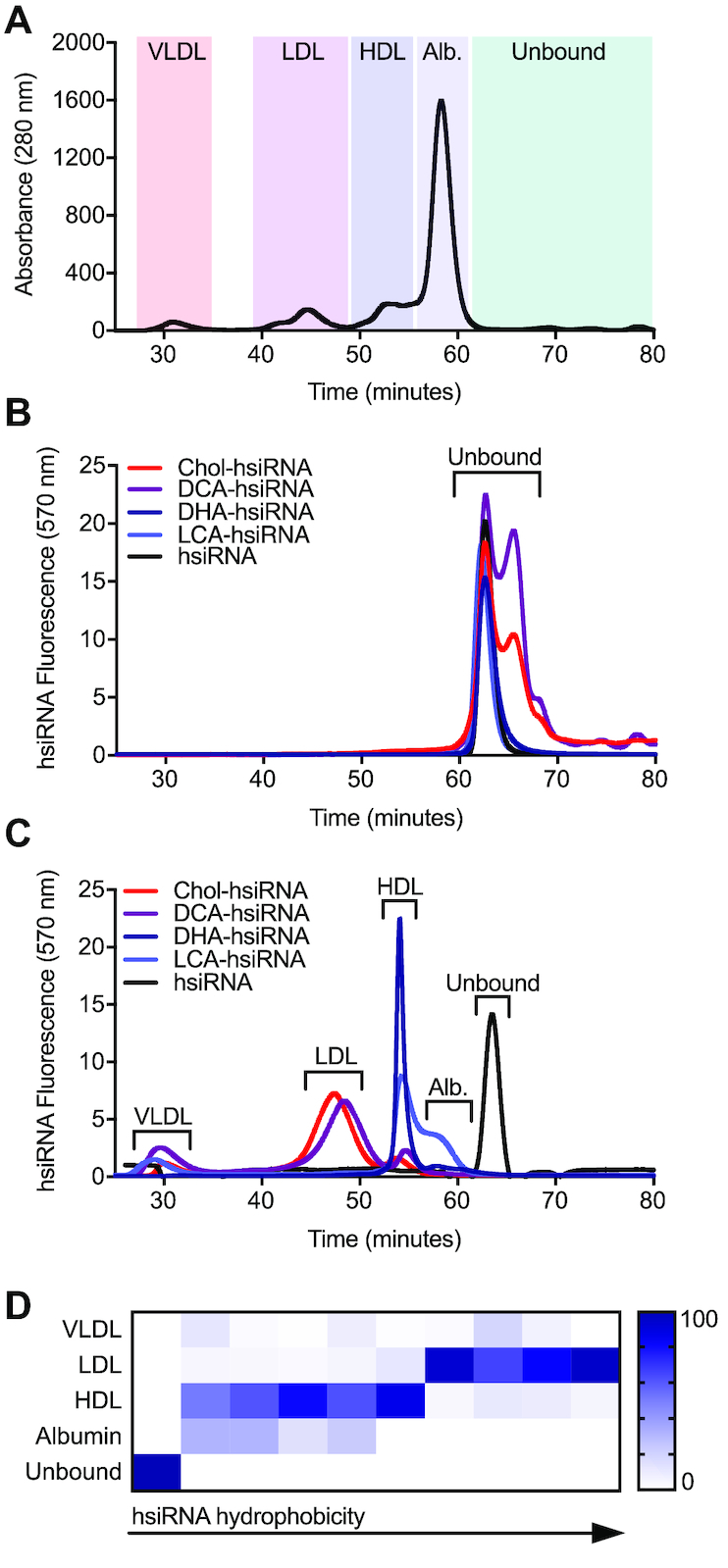
Lipoprotein binding profiles of lipid-conjugated hsiRNAs. (**A**) Mouse serum protein distribution following size exclusion chromatography (SEC). Red shading: VLDL; purple shading: LDL; dark-blue shading: HDL; light-blue shading: albumin; green shading: no protein. (**B**) Retention times of Cy3-labeled lipid-hsiRNAs following SEC. (**C**) Average retention times of Cy3-labeled lipid-hsiRNAs in mouse serum, 15 minutes after IV injection (*n* = 2). Peak shifts indicate serum protein association. (**D**) Summary of peak integrations of lipoprotein binding profiles for a variety of lipid-hsiRNAs.

To more precisely characterize the behavior of lipid-conjugated siRNAs in the circulation, we used high-resolution size exclusion chromatography (SEC) to measure the interaction between oligonucleotides and circulating proteins in mouse serum. Previous reports have suggested that sterol-, bile salt-, and fatty acid-siRNAs are capable of associating with both albumin and lipoproteins in the bloodstream (6). Thus, we used SEC to define relative size zones associated with very low-density lipoprotein (VLDL), low-density lipoprotein (LDL), and high-density lipoprotein (HDL) particles, in addition to small proteins (predominantly albumin) from mouse serum (Figure 3A). Peak assignments were confirmed by measuring cholesterol content in isolated fractions (Supplementary Figure S2). Assessment of the serum SEC profiles of wild-type (FVB/NJ) male and female animals (Supplementary Figure S2) revealed no apparent sex-dependent differences in lipoprotein peak assignment. Therefore, we performed subsequent analyses in female animals only.

To determine if lipid-hsiRNAs form stable complexes with lipoprotein particles or albumin *in vivo*, we first measured baseline retention times of free Cy3-labeled unconjugated and lipid conjugated-hsiRNAs (Figure 3B). The unbound forms of unconjugated hsiRNA, LCA-hsiRNA, and DHA-hsiRNA eluted at 62.9 min as a single peak. Unbound DCA-hsiRNA and Chol-hsiRNA eluted as a doublet, with peaks at both 62.9 and 65.8 min. The presence of a doublet may reflect a propensity for these two hydrophobic species to form reversible macromolecular structures under non-denaturing conditions. Next, we analyzed serum samples from mice injected intravenously with Cy3-labeled Chol-siRNA, DCA-siRNA, DHA-siRNA, LCA-siRNA or unconjugated siRNA (*n* = 2, 20 mg kg^−1^). Given the average elimination half-lives of Chol-hsiRNA and unconjugated hsiRNA (22), we collected serum 15 min after injection. By monitoring the baseline shift in hsiRNA fluorescence signal, we determined that each lipid conjugate has a unique lipoprotein binding signature *in vivo* (Figure 3C). In direct contrast to our *in vitro* results, we discovered that lipid-hsiRNA conjugates do not significantly associate with albumin in serum, likely due to the presence of higher affinity chaperones, such as lipoproteins. Unconjugated hsiRNAs did not associate with any serum components and were rapidly excreted into the urine (Figures 2A and 3C). LCA-hsiRNA associated predominantly with HDL (50.4% of total binding) and albumin (32.2%), with residual binding towards VLDL (12.2%) and LDL (5.3%). DHA-hsiRNA exhibited a stronger affinity for HDL (80.5%), with lesser co-migration with albumin (14.9%), LDL (3.7%) and VLDL (1%) peaks. Conversely, DCA-hsiRNA and Chol-hsiRNA were predominantly bound to LDL (65% and 82%, respectively), with residual binding to VLDL and HDL. Peak integrations (Figure 3C) are summarized in Supplementary Table S3.

To further investigate the similarity between the lipoprotein binding profiles and degree of hydrophobicity of siRNAs, we employed the same SEC methodology to analyze an expanded panel of lipid-hsiRNA conjugates. As described previously, we performed SEC-based profiling of lipoprotein-hsiRNA binding and plotted these data as a function of relative lipid-hsiRNA hydrophobicity (Figure 3D; Supplementary Table S3; Biscans *et al.*, 2018, NAR in this issue). We observed a strong correlation between hsiRNA hydrophobicity and segregation into albumin, HDL, and LDL peaks. Our findings suggest that oligonucleotide hydrophobicity can be engineered to achieve predictable and stepwise partitioning into different lipid transport pathways *in vivo*.

### Lipid-hsiRNAs elicit sustained systemic *in vivo* gene silencing after a single injection

Given that lipid-hsiRNAs predominantly complexed with circulating lipoproteins after systemic administration, we assessed the cell-specific distribution and gene-silencing efficacy of lipid-hsiRNAs in tissues that are enriched in lipoprotein receptors. For all tissues, cell-specific distribution of hsiRNAs was measured 48 h after a single, subcutaneous injection into mice (*n* = 3, 20 mg kg^−1^). Gene-silencing efficacy was determined by quantifying *Ppib* mRNA levels one week after a single, subcutaneous injection of *Ppib*-targeting hsiRNAs (*n* = 6, 20 mg kg^−1^).

#### Liver

**Figure 4.**
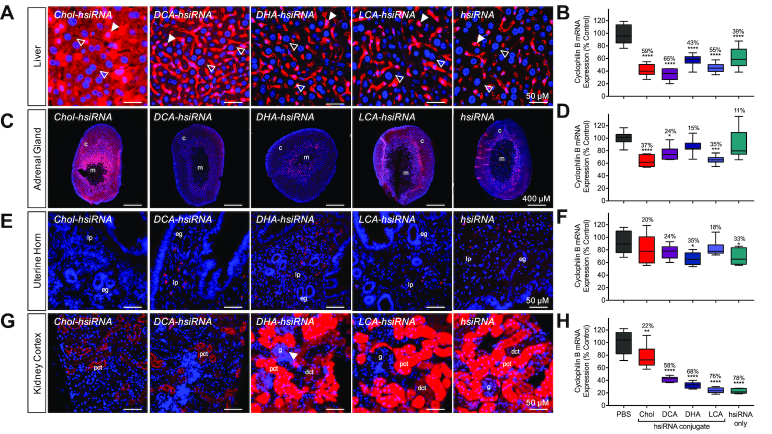
Distinct cellular uptake and efficacy patterns of lipid-conjugated hsiRNAs. Tissue-dependent internalization of lipid-hsiRNAs 48 h after a single, subcutaneous injection (*n* = 3 mice, 20 mg kg^−1^) in (**A**) liver, (**C**) adrenal gland, (**E**) uterine horn and (**G**) kidney cortex. Cy3-labeled lipid-hsiRNAs (red), nuclei stained with DAPI (blue). Arrowheads described in text. c: cortex; m: medulla; lp: lamina propria; eg: endometrial gland; pct: proximal convoluted tubule; dct: distal convoluted tubule; g: glomerulus. Quantification of *Ppib* silencing by non-labeled lipid-hsiRNAs in (**B**) liver, (**D**) adrenal gland, (**F**) uterine horn and (**H**) kidney cortex. *Ppib* mRNA levels were measured with QuantiGene 2.0 (Affymetrix) assay and normalized to a housekeeping gene, *Hprt*. All data presented as percent of saline-treated control. All error bars represent mean ± SD. *P < 0.05; **P < 0.01; ***P < 0.001; ****P < 0.0001 as calculated by one-way ANOVA with Tukey's test for multiple comparisons.

LDL is the principal intercellular transporter of cholesterol, and LDL receptors (LDLR) are abundantly expressed on liver hepatocytes, adrenal cortex, bronchial epithelial cells, and adipocytes. We first monitored cell tropism in the liver and found that LDL-associated oligonucleotides (Chol- and DCA-hsiRNA) were readily visualized within cytoplasmic foci in liver hepatocytes (Figure 4A, open arrows), while HDL- and albumin-associated hsiRNAs (DHA-, LCA-, and unconjugated hsiRNA) were undetectable (Figure 4A, open arrows). By contrast, we did observe oligonucleotide internalization in Kupffer cells (stellate macrophages) for all five hsiRNAs (Figure 4A, closed arrows). This result is consistent with what is known regarding the mononuclear phagocyte (reticuloendothelial) system as a first response against exogenous substances, including siRNAs and antisense oligonucleotides. We confirmed our findings for Chol-hsiRNA, DCA-hsiRNA, and DHA-hsiRNA by FACS, noting hsiRNA accumulation in 28–59% percent of endothelial cells and 18–49% of Kupffer cells, respectively (*n* = 3, 10 mg kg^−1^, Supplementary Figure S3). Next, we correlated hsiRNA accumulation with *Ppib* mRNA levels. As expected, Chol-hsiRNA and DCA-hsiRNA displayed the highest activity, reducing liver *Ppib* mRNA expression by 59% and 65%, respectively (Figure 4B, Supplementary Table S4). Unconjugated hsiRNA reduced *Ppib* mRNA levels by 39% (Figure 4B). We did not observe a reduction in *Ppib* mRNA after administration of non-targeting control hsiRNAs (*n* = 6, 20 mg kg^−1^, Supplementary Figure S4). We also did not observe significant upregulation of liver toxicity biomarkers, alkaline phosphatase (ALP) and alanine aminotransferase (ALT), in the blood (Supplementary Figure S5).

#### Adrenal gland

Circulating lipoproteins contribute over half of the cholesterol required for steroidogenesis in adrenocortical cells (24). Therefore, we wanted to assess the pattern of lipid-hsiRNA distribution within the adrenal gland. As a general trend, higher levels of lipid-hsiRNA fluorescence were observed in the adrenal cortex compared to the medulla (Figure 4C). The sterol-conjugated hsiRNAs (Chol and LCA) presented the highest levels of internalization in the adrenal cortex (Figure 4C), and the strongest activity. Chol-hsiRNA silenced *Ppib* mRNA up to 37%, while LCA-hsiRNA reduced *Ppib* mRNA levels by 35% (Figure 4D, Supplementary Table S4). DCA-, DHA-, and unconjugated hsiRNA showed lower levels of internal fluorescence in the adrenal cortex, and induced 24%, 15% and 11% *Ppib* silencing, respectively (Figure 4D).

#### Uterus

HDL is an integral component of the reverse cholesterol transport pathway, which transports lipids from extrahepatic tissues to the liver for excretion or recycling. Lipid transfer is commonly mediated by scavenger receptor B1 (SR-BI), which is highly expressed in liver sinusoidal endothelial cells, adrenal gland, ovary, and testis. Therefore, we monitored lipid-hsiRNA accumulation and activity within the uterus of female mice. DCA-, DHA-, LCA- and unconjugated hsiRNAs showed punctate staining throughout the lamina propria, with minimal staining of the endometrial glands (Figure 4E). We measured significant levels of *Ppib* silencing for both DHA-hsiRNA (35%) and unconjugated hsiRNA (33%) in biopsies from the uterine horn (Figure 4F, Supplementary Table S4).

#### Kidneys

LCA-, DHA- and unconjugated hsiRNAs were distributed similarly within the kidney cortex, staining both proximal and distal convoluted tubules (Figure 4G). Minor levels of fluorescence in the glomerulus were detected for all three oligonucleotide conjugates, with DHA-hsiRNA showing the most profound staining within the Bowman's capsule (Figure 4G, closed arrow). Chol-hsiRNA and DCA-hsiRNA were detected primarily within proximal convoluted tubules (Figure 4G). After measuring *Ppib* mRNA levels in biopsies from the kidney cortex, we noted parallels between accumulation and activity, with unconjugated hsiRNAs showing the highest level of silencing (78%) (Figure 4H, Supplementary Table S4). We did not observe any variability in biomarkers of kidney toxicity, including blood urea nitrogen and creatinine levels (Supplementary Figure S5). We also monitored other standard serum toxicological markers, including electrolytes (Ca^2+^, phosphate, Na^+^, K^+^), albumin, globulin, bilirubin, and glucose, and saw no significant changes in total concentration after hsiRNA, DHA-hsiRNA, or DCA-hsiRNA treatment (Supplementary Figure S5).

Taken together, these data suggest that lipid-conjugated hsiRNAs engage distinct lipid transport pathways and elicit gene silencing in a variety of tissues with minimal systemic toxicity. Interestingly, in some tissues, we observed a non-linear relationship between hsiRNA tissue accumulation and silencing efficacy, suggesting the presence of both productive and non-productive internalization pathways. This has been described for phosphorothioate-modified antisense oligonucleotides, which are intrinsically more active in hepatocytes than in the nonparenchymal cells of the liver (25). The observed discrepancy between levels of hepatocyte internalization after 48 h and mRNA silencing after 1 week may indicate translocation of hsiRNAs from sinusoidal depots into hepatocytes, or the presence of inherently more potent endocytic pathways in hepatocytes (Figure 4A and 4B). Whether this extends to the kidney or other tissues is unknown.

### hsiRNAs are internalized independently of LDL recycling via the LDL receptor

**Figure 5.**
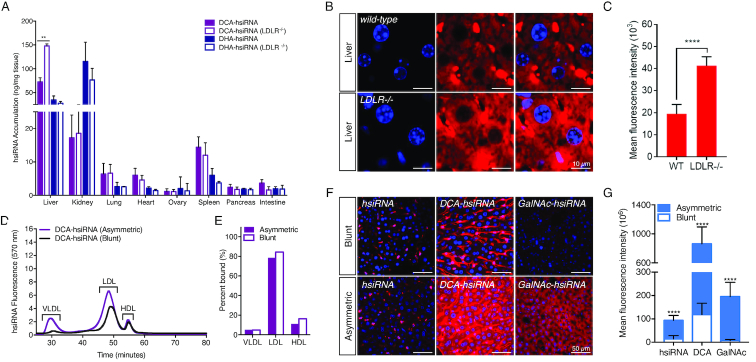
Mechanistic analysis of lipid-hsiRNA internalization in liver. (**A**) Guide strand quantification of Cy3-labeled DHA-hsiRNAs and DCA-hsiRNAs in wild-type (C57BL/6J) and LDLR deficient animals after a single, intravenous injection (*n* = 3 mice, 10 mg kg^−1^) using a PNA hybridization-based assay. Data presented as mean ± SD. (**B**) Hepatocyte internalization of Cy3-labeled DCA-hsiRNA in wild-type and LDLR deficient animals after a single, intravenous injection (n = 3 mice, 10 mg kg^−1^). Image is representative. Cy3-labeled DCA-hsiRNAs (red), nuclei stained with DAPI (blue). (**C**) Quantification of fluorescent signal from images acquired in (B). (**D**) Average retention times of Cy3-labeled DCA-hsiRNAs in mouse serum, 15 minutes after IV injection (*n* = 2 mice, wild type or LDLR^−/−^). (**E**) Average peak integrations from lipoprotein profiles in (D). (**F**) Hepatocyte internalization of Cy3-labeled blunt and asymmetric siRNAs (unconjugated, DCA-conjugated, or GalNAc-conjugated) after a single, subcutaneous injection (*n* = 3 mice, 20 mg kg^−1^), staining as described in (B). (**G**) Quantification of fluorescent signal from images acquired in (F).

To probe the dependency of hsiRNA internalization on lipoprotein receptor expression, we compared the systemic biodistribution of an LDL-associated hsiRNA (DCA-hsiRNA) and an HDL-associated hsiRNA (DHA-hsiRNA) in both wild-type and LDLR-deficient mice. We hypothesized that if LDL-associated hsiRNAs are dependent on LDL endocytosis for internalization, their biodistribution should be significantly perturbed by LDLR depletion. LDLR-dependent internalization into the liver has been described for both cholesterol- and α-tocopherol-conjugated duplex siRNAs (6,26). We intravenously injected wild-type (C57BL/6J) and LDLR-deficient (LDLR^−/−^) mice with DHA- or DCA-hsiRNA and quantified tissue accumulation by PNA hybridization (*n* = 3, 10 mg kg^−1^). As expected, DHA-hsiRNA systemic distribution and tissue internalization remained unchanged between wild-type and LDLR^−/−^ animals (Figure 5A, blue bars). Unexpectedly, DCA-hsiRNA liver accumulation increased two-fold in the absence of LDLR (Figure 5A, purple bars). This finding suggests that LDL-associated oligonucleotides are internalized in the liver by a mechanism independent of lipoprotein endocytosis. To determine if the observed increase in liver accumulation was cell-type specific, we acquired high-resolution images of the liver following subcutaneous administration of Cy3-labeled DCA-hsiRNAs (*n* = 2, 10 mg kg^−1^). Hepatocyte fluorescence in LDLR^−/-^ mice was visibly increased compared to wild-type mice (Figure 5B). Image quantification revealed a two-fold increase in fluorescence in mutant animals (Figure 5C). Taken together, these data suggest that hepatocyte internalization of LDL-associated hsiRNAs can occur independently of LDL endocytosis through LDLR.

### hsiRNA hepatocyte internalization is facilitated by a phosphorothioate-modified, single-stranded overhang

A defining feature of the hsiRNA constructs used in this study is the presence of a 5-nt single-stranded, phosphorothioate-modified (PS) overhang, which resembles the fully PS, single-stranded backbone of conventional antisense oligonucleotides (ASOs). Recent studies investigating ASO internalization by hepatocytes have established that the asialoglycoprotein receptor (ASGPR) contributes to the uptake of unconjugated PS ASOs (27,28). Therefore, one of the hepatocyte uptake pathways for DCA-hsiRNA may be a receptor-mediated process that requires a single-stranded, PS-modified overhang. To probe the structural requirements for hepatocyte uptake, we compared the asymmetric DCA-hsiRNA construct used throughout this study with a 20-nt blunt-ended DCA-conjugated hsiRNA. After analyzing the lipoprotein binding profiles of the asymmetric and blunt DCA-hsiRNAs, we observed no differences in lipoprotein binding character (Figure 5D). Similar to DCA-hsiRNA, blunt DCA-hsiRNA primarily associated with LDL (84.3%) and exhibited less affinity for HDL (11.1%) and VLDL (4.8%) (Figure 5E). This result provides further confirmation that lipoprotein association is mediated by the lipid conjugate and suggests that these compounds have comparable elimination half-lives *in vivo*. However, following a single, subcutaneous injection of Cy3-labeled blunt or asymmetric DCA-hsiRNA in wild-type mice (*n* = 2, 20 mg kg^−1^), we observed significantly less fluorescent signal in hepatocytes for blunt DCA-hsiRNA (Figure 5F). This suggests that the single-stranded PS overhang contributes significantly to hepatocyte uptake. To determine whether the presence of a single-stranded PS region is sufficient for unconjugated hsiRNA internalization, we compared the liver distribution pattern of blunt and asymmetric unconjugated hsiRNAs following administration to mice (*n* = 2, 20 mg kg^−1^). Although liver exposure of unconjugated hsiRNAs is limited, due to its rapid elimination half-life, we still observed a significant increase in hepatocyte fluorescence of asymmetric compared to blunt hsiRNA (Figure 5F, G). To further validate our finding that single-stranded PS modifications enhance hepatocyte uptake, we synthesized triantennary GalNAc-conjugated blunt and asymmetric hsiRNAs. GalNAc-conjugated siRNAs are specifically and rapidly internalized in hepatocytes through the ASGPR receptor. Here, we detected a marked increase in GalNAc-mediated hepatocyte internalization in the presence of a single-stranded PS tail (Figure 5F, G). The asymmetric compounds containing phosphodiester rich tail have reduced liver uptake and efficacy (data not shown), suggesting that phosphorothioates, rather than structure, principally contribute to enhance internalization. However, this data should be interpreted with caution—a decrease in phosphorothioate content negatively impacts the overall stability of oligonucleotides, which negatively affects activity. While the amount of siRNA accumulation was dramatically enhanced by the presence of the phosphorothioated tail, the blunt compounds were similarly functional *in vivo* (29). This finding suggests that phosphorothioate-enhanced internalization may not contribute significantly to productive uptake, at least in the short term. This is consistent with most double-stranded GalNAc siRNAs conjugates that show robust efficacy in the clinic (30,31).

## DISCUSSION

Here we demonstrate that changing the lipophilic modality attached to the fully chemically stabilized hsiRNA have a profound impact on the hsiRNA tissue distribution profile. In general, oligonucleotides tend to distribute to tissues with filtering function, with higher blood flow and discontinues, fenestrated endothelium: liver, spleen and kidneys (2). Hydrophobically modified hsiRNAs, in general follow these rules, with liver, spleen and kidney being primary tissues for distribution. Although, the exact pattern of distribution and relative liver/kidney ratio is heavily affected by the nature of the conjugate. The more hydrophobic conjugates (cholesterol, DCA) preferentially deliver to the liver. The less hydrophobic (DHA, LCA) conjugate shows much lower liver accumulation and preferentially distribute to kidneys.

Unconjugated hsiRNAs are quickly cleared by the kidneys (>85%) from the blood stream with a serum half-life of a couple of minutes (32) (Biscans *et al*, 2018, NAR, in this issue). The retained compounds mostly accumulate in the kidney proximal tubule epithelia, likely due to entrapment during filtration. The compounds used in this study contain ∼30% phosphorothioate (PS) modifications (13 PS bonds out of 35 linkages). The kidney epithelia retention is fully dependently on the presence of the phosphorothioates. Fully stabilized, non-PS modified hsiRNAs are cleared semi-quantitatively with minimal levels of kidney or any other tissue retention and are not detectable four hours post injection (33,34). It is likely, that the observed PS-dependent proximal tubule retention is similar in mechanism to antisense kidneys distribution. Short ASOs (12-16 bases, fully phosphorothioated) preferentially accumulate in kidneys, which is a strategy that has been considered extensively for ASO-based modulation of gene expression in kidney diseases (35). After intravenous or subcutaneous administration, unconjugated hsiRNAs are rapidly filtered in the kidneys from the blood into the glomerular space, where they are reabsorbed (likely via scavenger receptors) by proximal tubule cells or excreted through the urine. Despite their short residence time, unconjugated hsiRNAs achieve ∼80% target mRNA silencing in the kidney (Figure 4), providing a simple strategy for modulation of gene expression in proximal tubule epithelia (14). Interestingly, the level of kidney silencing can be affected by presence of the hydrophobic fluorescent label, which negatively impacts silencing (Biscans *et al.*, 2018, NAR, in this issue).

The circulation half-life for lipid-conjugated hsiRNA is significantly increased (32) likely due to spontaneous association with circulating lipoproteins. This increases plasma half-life and promotes exposure to extra renal tissues. Different conjugates have a different impact on circulation half-life with more lipophilic compounds having slower clearance kinetics (32). Here, we explore if the observed changes in both clearance and tissue distribution profiles can be explained by changes in proteins in serum associated with different type of hsiRNAs. Indeed, we observe that more hydrophobic hsiRNAs (Cholesterol, DCA) preferentially bind LDL, while relatively less hydrophobic hsiRNAs (DHA, LCA) associate more with HDL and non-conjugated hsiRNAs show limited serum binding.

The lack of significant serum protein association of non-conjugated hsiRNA makes sense. Any minimal observed retention is defined by phosphorothioates. It is well established that both minimal number of phosphorothioate and PS/PO ratio defines serum protein association of ASOs (2). It is believed that a minimum of 16–18 PS in a row is necessary to promote serum association, and thus liver delivery, and shorter ASOs or ASOs with lower PS content show preferential kidney accumulation (20). The compounds used in this study have only 13 PS bonds which represent ∼30% of nucleotide linkages, and thus are not expected to display significant serum binding.

The mouse plasma is significantly enriched in HDL compared to LDL (6). In spite of that, chol-hsiRNAs, preferentially associate with LDL indicating a much tighter binding affinity. The serum binding profile was evaluated at 15 minutes post injection (∼half clearance rate, (32)). Interestingly, Wolfrum *et al.* (6) showed similar affinity of chol-modified siRNAs to LDL and HDL, although in a context of purified plasma *in vitro*. The differences in observed profiles are likely related to the differences between *in vitro* and *in vivo* steady state conditions.

In general, LDL-associated hsiRNAs enter into the endogenous lipid transport pathway and are preferentially internalized in LDL receptor-enriched tissues, such as liver, lung, and intestine. HDL-associated hsiRNAs are transported via reverse cholesterol transport and exhibit enhanced uptake and efficacy in SR-BI-enriched tissues, such as adrenal gland and ovary. Moreover, the lipid transport pathway engaged by lipid-conjugated hsiRNAs may be determined *a priori* following measurement of lipid-siRNA hydrophobicity. Thus in general, hydrophobicity correlates with serum binding profile with more hydrophobic siRNAs associated with LDL and less hydrophobic with HDL, which in parts predicts, to a significantly degree, tissues distribution profile, or at least liver/ kidney uptake ratio.

VLDL and LDL carry highly-nonpolar saturated triglycerides and unsaturated cholesteryl esters, respectively, while HDL carries cholesterol and unsaturated phospholipids (relatively more polar). hsiRNAs equilibrate with circulating lipoproteins based on the solubility of the lipid conjugate in the lipoprotein core. A hypothesis that has been independently corroborated by the observation that even when highly-lipophilic Chol-siRNA conjugates are exogenously loaded into HDL, they rapidly redistribute into LDL in serum (6).

Several recent studies have postulated that lipid-conjugated oligonucleotides leverage plasma albumin as an endogenous carrier (7,36). Lipid-conjugated hsiRNAs showed minimal binding to albumin *in vivo* (Figure 3), despite robust affinity in *vitro* (Figure 3). This difference between *in vitro* and *in vivo*, is again easily explained by the relative affinities model. Lipid-conjugated siRNAs bind tightly to purified albumin *in vitro*. *In vivo*, affinity to LDL is significantly higher resulting in preferential binding and no albumin distribution. Most prior studies were done at 10 and 20 mg/kg dose levels, which is likely below the LDL binding capacity and some albumin binding might be observed at much higher dose levels when both LDL and HDL will be saturated.

What is the role of LDL in lipophilic siRNA trafficking: distribution, uptake or both? To answer this question, we evaluated distribution of lipid-conjugated siRNA in LDLR-deficient animals (37). We observed that both clearance and tissue distribution profiles were almost identical. On top of it, LDLR-deficient animals showed ∼two-fold enhancement in liver accumulation. Thus, it is likely that while LDL binding is essential for clearance and distribution, it is not a primary factor driving hsiRNA internalization.

The conclusion that lipoprotein receptors do not principally contribute to lipophilic siRNA cellular internalization is consistent with previously reported data. Wolfrum *et al.* demonstrated that the half clearance rate of cholesterol-conjugated siRNAs was markedly faster (hours vs days) than LDL and HDL (6). Both data sets are consistent with the model, where lipoprotein-bound cholesterol-siRNAs are carried by LDL and HDL particles, but primarily internalized by an independent mechanism. There is a body of literature suggesting that scavenger receptors may be involved in cholesteryl ester internalization in LDL-R negative mice (38), potentially explaining the observed (in LDLR mutant) two-fold increase in lipophilic siRNA tissue uptake.

Is hydrophobicity the only factor driving distribution and tissue accumulation? In general, the correlation between distribution and accumulation is great, it is not absolute. Cholesterol and DCA show very similar hydrophobicity and serum binding profiles with preferential LDL association. While liver accumulation for both conjugates were similar, extrahepatic delivery observed with DCA was significantly higher than with cholesterol (up to 7-fold more in lung and heart). These findings may be due to a tissue-specific internalization mechanism and is a subject of further investigation (see more in Biscans *et al.*, 2018, NAR, in this issue). It is possible that the identity of the conjugate may contribute to cell surface adsorption, where internalization may occur through an independent mechanism as hypothesized by Crooke *et al.* (39). In addition, the nature of the conjugate may impact efficiency of endosomal escape, potentially explaining observed functional differences between lipophilic conjugates showing similar tissue accumulation. (Biscans *et al.* for details, 2018, NAR, in this issue).

In this study we used fully chemically stabilized asymmetric phosphorothioated (∼30%) siRNAs. Full chemical stabilization is essential for conjugate mediated delivery (8) and clinically advanced GalNAc conjugate compounds are extensively stabilized (4,27,40). Cholesterol has been explored as a delivery modification for both blunt and asymmetric compounds with conceptually similar pharmacokinetic behavior (33,41). With that said, phosphorothioates are a primary force behind ASO delivery and significantly enhances cholesterol mediated uptake in a mechanism similar to that of ASOs (27,28).

To evaluate the relative impact of PS modifications (∼30% content) on a conjugate mediated delivery *in vivo*, we compared serum binding, distribution of blunt and asymmetric fully chemically modified siRNAs conjugated to GalNAc and DCA. While the overall serum binding profile was similar, PS enriched siRNAs showed significantly higher accumulation in liver independently of the presence or nature of the conjugate. The liver accumulation was 10 to 100-fold higher in the context of PS-enriched asymmetric compounds. These findings suggest that both lipoprotein endocytosis and phosphorothioate modifications may contribute to liver accumulation and cellular internalization cooperatively. It is likely that LDL and HDL binding is essential for a slower clearance rate and enabling liver distribution, while phosphorothioate modifications principally contribute to cellular internalization. This observation is consistent with previously reported cooperativity in liver internalization between GalNAc and PS modifications (4,27,40).

It is important to note that while the level of accumulation of the GalNAc-conjugate blunt siRNAs was lower than asymmetric, it was sufficient to induce highly potent silencing, consistently with most of clinically-active GalNac conjugated-siRNAs being symmetric (4,27,40). In spite of functional efficacy, we were surprised to see such a low level of GalNAc-blunt siRNA liver accumulation. Most of the clinical compounds are not blunt but carry a two base-pair phosphorothioated overhang (42). It is possible that these short PS-modified overhang might be functionally contributing to hepatocytes internalization. It is also important to note, that while GalNac blunt accumulates in liver exclusively, GalNAc asymmetric PS-enriched compounds deliver equally well to liver and kidneys (43). Thus, for selective liver targeting symmetric siRNA design might be preferred.

One of the interesting recent direction is the exploration of biomimetic lipoprotein nanoparticles as siRNA delivery vehicles (44–49). Indeed, pre-formulation of cholesterol-conjugated siRNAs with HDL enhanced in vivo efficacy 8- to 15-fold (44,45,50). In this study, partially modified siRNAs were used, which are inherently unstable in serum (51,52). It is likely that the observed enhancement in activity was mostly due to stabilization of the siRNA in a protein complex.

In the context of using fully chemically-stabilized hsiRNAs, complexing happens almost instantaneously *in vivo*, thus harnessing endogenous lipoproteins to slowdown clearance and enable wide tissue distribution. This approach allows use of chemically-defined entities, oligonucleotides directly and circumvents the typical challenges associated with synthetic nanoparticles, including low loading efficiency and yield (53), complex surface-modification processes, and systemic toxicity (54).

Finally, an important advantage of lipid-conjugation is that its impact on biodistribution is sequence-independent. Thus, this strategy should behave analogously for the delivery of other small RNA cargo, including miRNAs, antagomirs or CRISPR components. Although lipid conjugation as a delivery platform has many strengths, it also has limitations, chief among them lack of targeted, cell type-specific delivery. Thus, its likely applicability is limited to targets and indications where clinically relevant target expression is limited to targetable tissue and its modulation in clearance tissues like liver, kidney and spleen is not of concern, as has recently been demonstrated for modulation of sFLT1 in placenta (55) or myostatin in muscle (56).

## Supplementary Material

Supplementary Data
