# Supplementary material for: Hydrophobicity drives the systemic distribution of lipid-conjugated siRNAs via lipid transport pathways
Source: Nucleic Acids Res. 2018 Dec 8;47(3):1070–81. doi: 10.1093/nar/gky1232 (PMC6379714; doi:10.1093/nar/gky1232)
Supplement: Supplementary Data [file gky1232_supplemental_files.pdf]

Hydrophobicity drives the systemic distribution of lipid-conjugated siRNAs via lipid transport pathways

Maire F. Osborn[1,2,*,‡], Andrew H. Coles[1,2,*], Annabelle Biscans[1,2], Reka A. Haraszti[1,2], Loic Roux[1,2], Sarah Davis[1,2], Socheata Ly[1,2], Dimas Echeverria[1,2], Matthew R. Hassler[1,2], Bruno M.D.C. Godinho[1,2], Mehran Nikan[1,2,†], and Anastasia Khvorova[1,2,‡]

[1]RNA Therapeutics Institute, University of Massachusetts Medical School, Worcester, MA, USA
[2]Department of Molecular Medicine, University of Massachusetts Medical School, Worcester, MA, USA

Co-corresponding authors: Maire F. Osborn, Email: maire.osborn@umassmed.edu. Phone: (508) 654-6215. Anastasia Khvorova, Email: Anastasia.khvorova@umassmed.edu. Phone: (774) 455-3638.

[*]These authors contributed equally to this work
[†]Present address: Ionis Pharmaceuticals, Carlsbad, CA
[‡]Co-corresponding authors

Table of Contents

Supplementary Materials and Methods S2
Table S1. Oligonucleotide sequences and chemical modification patterns S3
Table S2. Predicted lipid partition coefficients S3
Figure S1. Lipid-hsiRNAs interact with albumin S4
Figure S2. Lipoprotein chromatography and cholesterol content in male and female plasma S5
Table S3. Summary of peak integrations from lipoprotein binding profiles S6
Figure S3. FACS profiling of lipid-hsiRNA accumulation in liver S7
Table S4. Full panel of statistics analysis for mRNA silencing levels in different tissues S8
Figure S4. mRNA silencing following administration of non-targeting controls S9
Figure S5. Lipid-hsiRNA treatment does not induce any changes in blood chemistry S9

Supplementary Materials and Methods

Gel shift assay

Lipid-hsiRNAs (5 µM) were incubated with increasing bovine serum albumin (0-200 µM or 0, 2, 5, 8, and 10 g/L) in 20 mM Tris pH 7.4. Samples was incubated for 17 h on an orbital shaker (100 rpm) at room temperature and subsequently analyzed by 6% native TBE EMSA. Bands were quantified using ImageJ software (ImageJ 1.50i) and affinity constants were calculated for DCA-hsiRNA and Chol-hsiRNA by determining the fraction of lipid-hsiRNA bound in each lane.

Fluorescence associated cell sorting (FACS)

Animals were injected subcutaneously (SC, interscapular, between shoulders) with 10 mg kg^{-1} of Cy3-labeled oligonucleotides (n = 3). After 1 week, mice were deeply anesthetized with 0.1% Avertin and livers were dissected and prepared for downstream analysis exactly as described previously[1]. hsiRNA, DCA-hsiRNA, and DHA-hsiRNA intracellular accumulation was measured in CD31+ endothelial cells and F4/80+ Kupffer cells by flow cytometry using an LSR II cell analyzer (Becton-Dickenson). Fluorescence-activated cell sorter analyses were prepared by FlowJo software (Tree Star Inc., Ashland, OR).

Toxicity

At the termination of the experiment described in Figure 4 (1 week after administration of test compounds), mouse blood (~500 µL) was collected in a sterile lithium heparin-coated tube following cheek incision with a lancet (n = 6). Samples were spun at 10,000 RPM for 10 minutes at 4°C to isolate serum. The UMMS National Mouse Metabolic Phenotyping Core analyzed the samples for the following parameters: blood urea nitrogen (BUN); alanine aminotransferase (ALT); alkaline phosphatase (ALP); albumin (ALB); amylase (AMY); calcium (Ca^{2+}); creatinine (CRE); globulin (GLOB); glucose (GLU); phosphorus (PHOS); potassium (K+); sodium (Na+); total bilirubin (TBIL), using an Abaxis comprehensive rotor.

Serum cholesterol measurement

For serum cholesterol quantification, whole mouse blood (~500 µL) was collected in a sterile EDTA-coated tube following cheek incision with a lancet from both male and female mice (n = 2). Samples were spun at 10,000 RPM for 10 minutes at 4°C. 50 µL of serum was directly injected on Superose 6 Increase 10/300 size exclusion column (GE Healthcare). Oligonucleotide migration was monitored by Cy3 fluorescence at 570 nm, and lipoprotein protein content was monitored by absorbance at 280 nm. Fractions were collected and analyzed for cholesterol content using the HDL and LDL Cholesterol Assay Kit (Abcam) according to the manufacturer's instructions.

siRNA ID	Gene	Accession Number	Targeting Position	Strand	Chemical Modification Pattern	Conjugate
hsiRNA ^{Ppib}	PPIB	NM_009693.2	437	S	fC#mA#fA.mA.fU.mU.fC.mC.fA.mU.fC.mG.fU#mG#fA	
Chol-hsiRNA ^{Ppib}	PPIB	NM_009693.2	437	S	fC#mA#fA.mA.fU.mU.fC.mC.fA.mU.fC.mG.fU#mG#fA	3'-Cholesterol
LCA-hsiRNA ^{Ppib}	PPIB	NM_009693.2	437	S	fC#mA#fA.mA.fU.mU.fC.mC.fA.mU.fC.mG.fU#mG#fA	3'-Lithocholic acid
DHA-hsiRNA ^{Ppib}	PPIB	NM_009693.2	437	S	fC#mA#fA.mA.fU.mU.fC.mC.fA.mU.fC.mG.fU#mG#fA	3'-Docosahexaenoic acid
DCA-hsiRNA ^{Ppib}	PPIB	NM_009693.2	437	S	fC#mA#fA.mA.fU.mU.fC.mC.fA.mU.fC.mG.fU#mG#fA	3'-Docosanoic acid
hsiRNA ^{Ppib}	PPIB	NM_009693.2	437	AS	VPmU#fU#mA.fA.mU.fC.mU.fC.mU.fU.mU.fA.mC#fU#mG#fA#mU#fA#mU#fA	
Cy3-hsiRNA ^{Ppib}	PPIB	NM_009693.2	437	S	fC#mA#fA.mA.fU.mU.fC.mC.fA.mU.fC.mG.fU#mG#fA	
Cy3-Chol-hsiRNA ^{Ppib}	PPIB	NM_009693.2	437	S	fC#mA#fA.mA.fU.mU.fC.mC.fA.mU.fC.mG.fU#mG#fA	3'-Cholesterol; 5'-Cy3
Cy3-LCA-hsiRNA ^{Ppib}	PPIB	NM_009693.2	437	S	fC#mA#fA.mA.fU.mU.fC.mC.fA.mU.fC.mG.fU#mG#fA	3'-Lithocholic acid; 5'-Cy3
Cy3-DHA-hsiRNA ^{Ppib}	PPIB	NM_009693.2	437	S	fC#mA#fA.mA.fU.mU.fC.mC.fA.mU.fC.mG.fU#mG#fA	3'-Docosahexaenoic acid; 5'-Cy3
Cy3-DCA-hsiRNA ^{Ppib}	PPIB	NM_009693.2	437	S	fC#mA#fA.mA.fU.mU.fC.mC.fA.mU.fC.mG.fU#mG#fA	3'-Docosanoic acid; 5'-Cy3
hsiRNA ^{NTC}	NTC	--	--	S	fU#mG#fA.mC.fA.mA.fA.mU.fA.mC.fG.mA.fU#mU#fA	
Chol-hsiRNA ^{NTC}	NTC	--	--	S	fU#mG#fA.mC.fA.mA.fA.mU.fA.mC.fG.mA.fU#mU#fA	3'-Cholesterol
LCA-hsiRNA ^{NTC}	NTC	--	--	S	fU#mG#fA.mC.fA.mA.fA.mU.fA.mC.fG.mA.fU#mU#fA	3'-Lithocholic acid
DHA-hsiRNA ^{NTC}	NTC	--	--	S	fU#mG#fA.mC.fA.mA.fA.mU.fA.mC.fG.mA.fU#mU#fA	3'-Docosahexaenoic acid
DCA-hsiRNA ^{NTC}	NTC	--	--	S	fU#mG#fA.mC.fA.mA.fA.mU.fA.mC.fG.mA.fU#mU#fA	3'-Docosanoic acid
hsiRNA ^{NTC}	NTC	--	--	AS	VPmU#fA#mA.fU.mC.fG.mU.fA.mU.fU.mU.fG.mU#fC#mA#fA#mU#fC#mA#fU	
Blunt Cy3-DHA-hsiRNA ^{Ppib}	PPIB	NM_009693.2	437	S	mA#fA#mC.fA.mG.fC.mA.fA.mA.fU.mU.fC.mC.fA.mU.fC.mG.fU#mG#fA	3'-Docosahexaenoic acid; 5'-Cy3
Blunt Cy3-DCA-hsiRNA ^{Ppib}	PPIB	NM_009693.2	437	S	mA#fA#mC.fA.mG.fC.mA.fA.mA.fU.mU.fC.mC.fA.mU.fC.mG.fU#mG#fA	3'-Docosanoic acid; 5'-Cy3
Blunt Cy3-GalNAc-hsiRNA ^{Pp}	PPIB	NM_009693.2	437	S	mA#fA#mC.fA.mG.fC.mA.fA.mA.fU.mU.fC.mC.fA.mU.fC.mG.fU#mG#fA	3'-N-acetylgalactosamine; 5'-Cy3
GalNAc-hsiRNA ^{Ppib}	PPIB	NM_009693.2	437	S	fC#mA#fA.mA.fU.mU.fC.mC.fA.mU.fC.mG.fU#mG#fA	3'-N-acetylgalactosamine; 5'-Cy3
Blunt hsiRNA ^{Ppib}	PPIB	NM_009693.2	437	AS	VPmU#fU#mA.fA.mU.fC.mU.fC.mU.fU.mU.fA.mC.fU.mG.fA.mU.fA#mU#fA	

Table S1. Modified oligonucleotide sequences. Chemical modifications are abbreviated as follows, where "X" corresponds to A, U, G, or C: fX (2'-fluoro), mX (2'-O-methyl), VP (5'-vinylphosphonate), '#' (phosphorothioate backbone modification), '.' (phosphodiester backbone). Oligonucleotide synthesis procedures are described in the Materials and Methods.

Molecule	Predicted LogP
Cholesterol	7.62
Docosanoic acid	9.13
Docosahexaenoic acid	5.68
Lithocholic acid	5.16

Table S2. Predicted partition coefficients for each lipid. logP values determined using the Molinspiration Interactive logP calculator (Molinspiration Property Calculation Service, www.molinspiration.com).

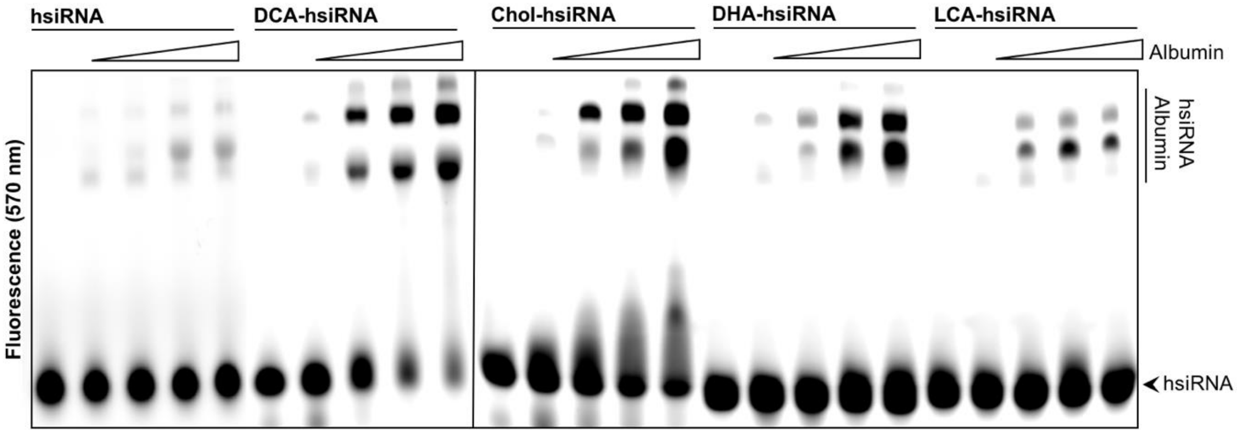

Figure S1. Lipid-hsiRNAs interact with albumin. Gel shift assay between lipid-hsiRNAs (5 μM) in the presence of increasing bovine serum albumin (0-200 μM, 0, 2, 5, 8, and 10 g/L).

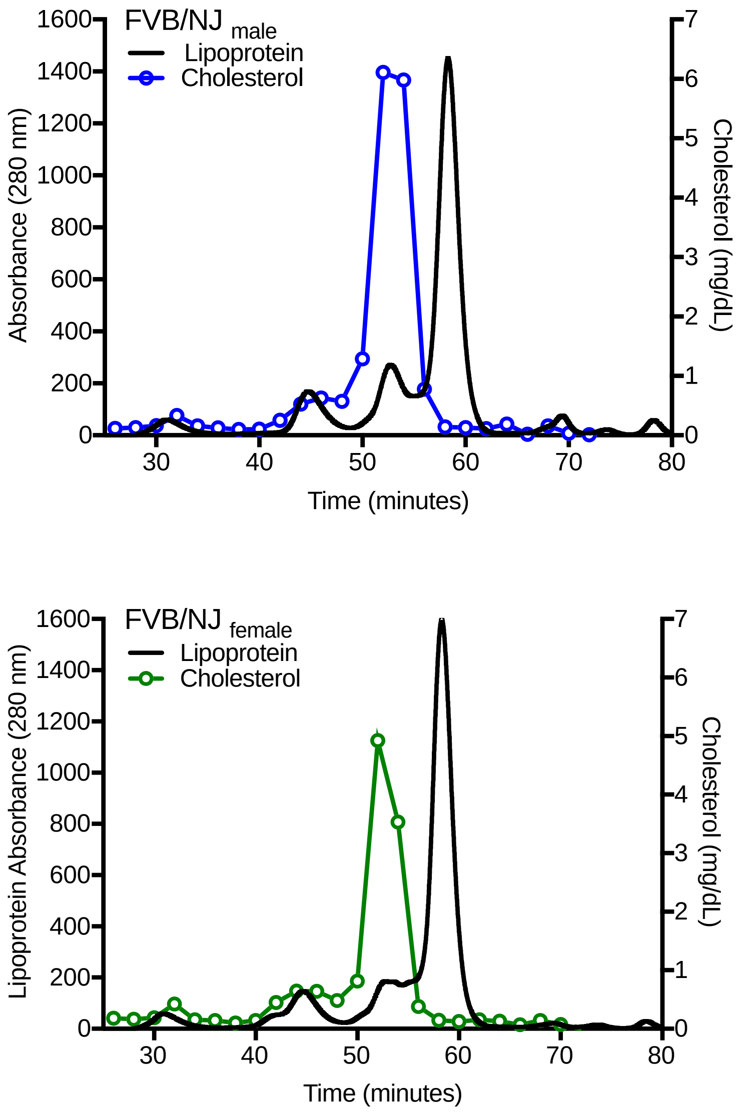

Figure S2. Lipoprotein and cholesterol profiling in male and female mouse plasma. Mouse serum lipoprotein and cholesterol distribution following size exclusion chromatography (SEC) from male (upper panel) or female (lower panel) wild-type animals. Black lines: serum proteins; blue/green lines: cholesterol.

	VLDL	LDL	HDL	Albumin	Unbound	Conjugate
hsiRNA	0.0	0.0	0.0	0.0	99.5	
LCA-hsiRNA	12.2	5.3	50.4	32.2	0.0	Lithocholic acid
PC-DHA-hsiRNA	2.9	5.0	60.5	31.7	0.0	Phosphocholine-docosahexaenoic acid
EPA-hsiRNA	9.3	6.1	61.6	23.0	0.0	Eicosapentanoic acid
DHA-hsiRNA	1.0	3.7	80.5	14.9	0.0	Docosahexaenoic acid
RA-hsiRNA	1.8	11.7	86.4	0.0	0.0	Retionic acid
PC-DCA-hsiRNA	2.7	92.2	4.9	0.0	0.0	Phosphocholine-docosanoic acid
DCA-hsiRNA	19.1	65.0	11.1	0.0	0.0	Docosanoic acid
Chol-hsiRNA	7.3	82.2	10.5	0.0	0.0	Cholesterol
GM1-hsiRNA	0.0	93.8	6.2	0.0	0.0	GM1 ganglioside

Table S3. Lipid-hsiRNAs partition into distinct LDL and HDL binding classes based on hydrophobicity. Lipoprotein binding profiles were collected for a series of lipid-hsiRNAs (as described in Figure 3). Peaks were auto-selected and integrated using Agilent ChemStation software.

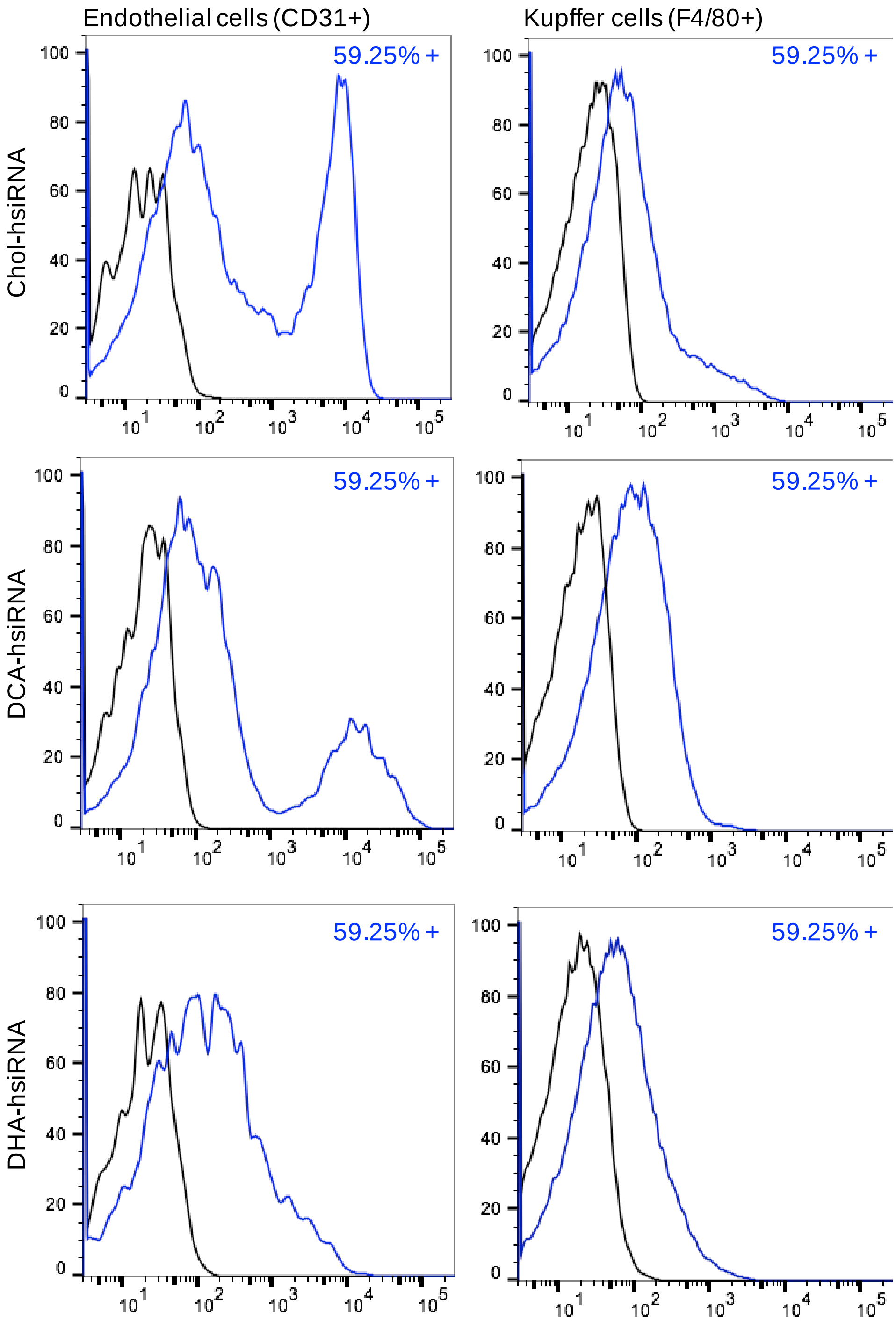

Figure S3. FACS profiling of lipid-hsiRNA accumulation in liver. hsiRNA, DCA-hsiRNA, and DHA-hsiRNA accumulation in CD31+ endothelial cells and F4/80+ Kupffer cells (stellate macrophages).

		PBS	Chol	LCA	DHA	DCA
Kidney Cortex	Chol	0.0013				
	LCA	0.0001	0.0001			
	DHA	0.0001	0.0001	0.62		
	DCA	0.0001	0.0001	0.03	0.55	
	hsiRNA	0.0001	0.0001	0.99	0.48	0.014
Liver	Chol	0.0001				
	LCA	0.0001	0.98			
	DHA	0.0001	0.08	0.33		
	DCA	0.0001	0.91	0.52	0.006	
	hsiRNA	0.0001	0.02	0.11	0.99	0.001
Uterine horn	Chol	0.66				
	LCA	0.83	0.99			
	DHA	0.02	0.43	0.25		
	DCA	0.37	0.99	0.97	0.7	
	hsiRNA	0.045	0.68	0.46	0.99	0.9
Adrenal gland	Chol	0.0001				
	LCA	0.83	0.99			
	DHA	0.02	0.43	0.25		
	DCA	0.37	0.99	0.97	0.7	
	hsiRNA	0.05	0.68	0.46	0.99	0.99

Table S4. Full panel of statistics analysis for mRNA silencing levels in different tissues

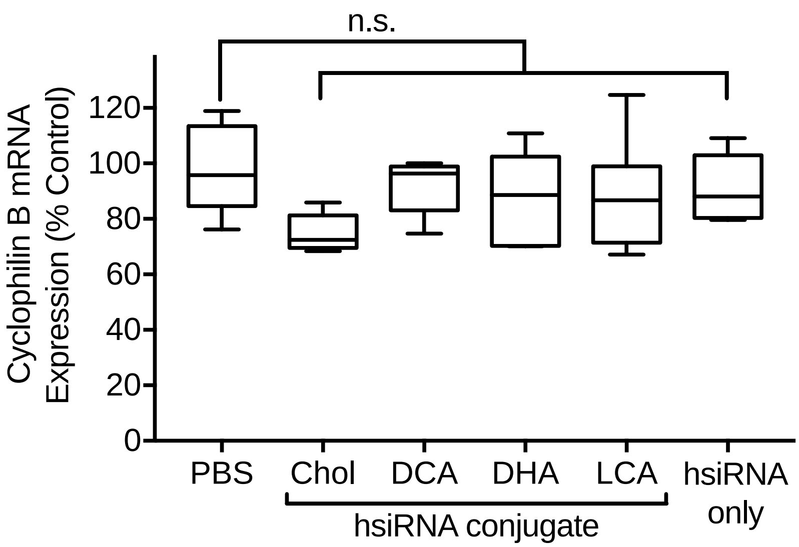

Figure S4. mRNA silencing following administration of non-targeting controls. Quantification of *Ppib* silencing by non-targeting control lipid-hsiRNAs in the liver. *Ppib* mRNA levels were measured with QuantiGene 2.0 (Affymetrix) assay and normalized to a housekeeping gene, *Hprt*. All data presented as percent of saline-treated control. All error bars represent mean ± SD. n.s. non-significant; *P < 0.05; **P < 0.01; ***P < 0.001; ****P < 0.0001 as calculated by one-way ANOVA with Tukey's test for multiple comparisons.

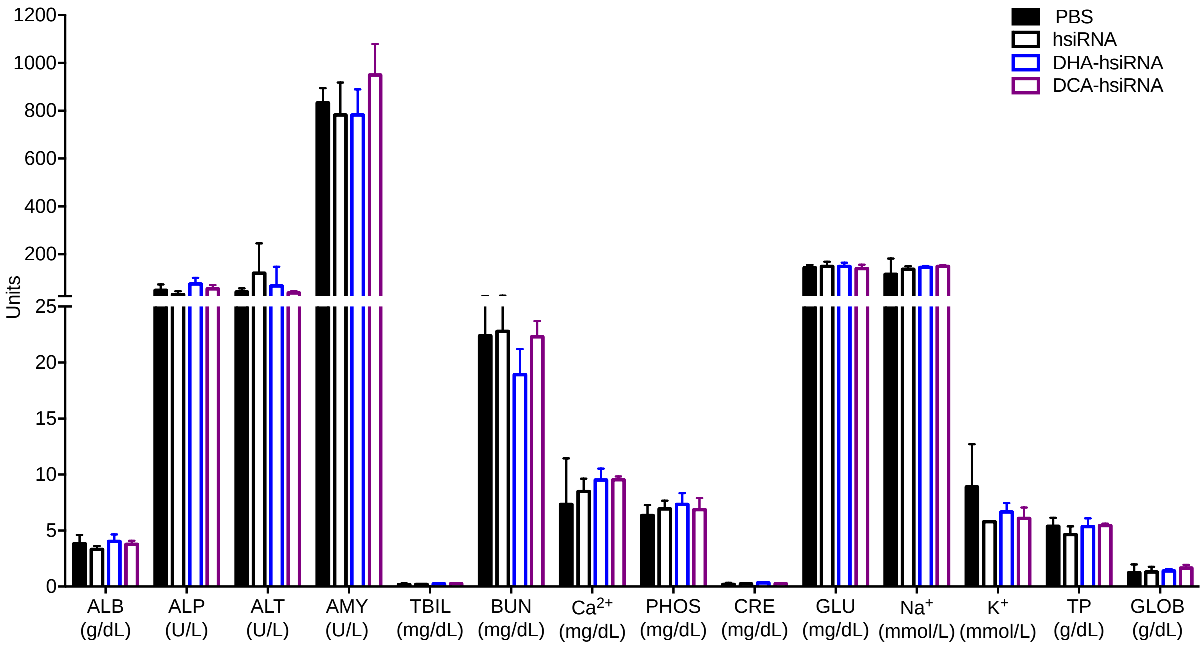

Figure S5. Lipid-hsiRNA treatment does not induce any changes in blood chemistry. A comprehensive panel of serum toxicological markers were measured one week after administration of test compounds (20 mg kg^{-1}, SC). No significant differences were observed between saline and hsiRNA-treated animals.

References

1 Severgnini, M. *et al.* A rapid two-step method for isolation of functional primary mouse hepatocytes: cell characterization and asialoglycoprotein receptor based assay development. *Cytotechnology* **64**, 187-195, doi:10.1007/s10616-011-9407-0 (2012).
